# A roadmap for human trials of xenotransplantation

**DOI:** 10.1172/JCI164484

**Published:** 2022-10-03

**Authors:** Paige M. Porrett, Jayme E. Locke

**Affiliations:** University of Alabama at Birmingham Heersink School of Medicine, Birmingham, Alabama, USA.

## A new era in transplantation

Kidney transplantation is the cure for end-stage kidney disease, but the overwhelming majority of patients who need a transplant will never get one, as more than 90,000 Americans wait and fewer than 25,000 kidney transplantations are performed each year (1). In fact, 17 Americans die each day waiting for an organ for transplantation (1). The transplantation of animal organs into humans has long been a logical solution to the organ shortage crisis, but the immunologic barriers to implementation of this solution have seemed insurmountable. Excitingly, several recent articles (2–4) suggest that an elusive goal of xenotransplantation has indeed been achieved: the generation of an animal organ source sufficiently humanized to avoid hyperacute rejection in human recipients. It almost seems like science fiction, but in reality, it’s the current science. As we blaze forward into a possible future liberated from the organ shortage, we must not lose sight of our guiding scientific principles. Although there is an immediate, immense scarcity of available organs, it is imperative that we hold fast to the foundation of 50 years of transplantation science and maintain recipient safety.

Human-to-human organ transplantation (i.e., allotransplantation) is an outstanding therapeutic modality that durably cures end-stage organ disease in highly selected patients. First-in-human trials of xenotransplantation are extremely unlikely to achieve the outcomes produced by decades of refinement in allotransplantation, nor should the field of xenotransplantation be expected to produce such outcomes in these early years. That said, translational scientists who are designing and implementing first-in-human clinical trials should adhere to well-established transplantation and scientific “first principles.” Adherence to such principles will minimize risk for first-in-human xenotransplant recipients and deliver xenotransplantation to the world as quickly as possible.

### Perform prospective compatibility testing.

The identification of graft-destructive antibodies and the creation of platforms to test for such antibodies have contributed largely to the success of contemporary allotransplantation. We know that compatibility is important in xenotransplantation as well, as preformed antibodies in human sera targeting carbohydrate antigens lead to hyperacute rejection. Even in the setting of xenotransplantation with gene-edited organs that lack the stimulating carbohydrate antigens, crossmatching is a necessity, as preformed HLA-specific antibodies in human recipients could cross-react with swine leukocyte antigen (SLA), given the significant homology between class II HLA and SLA. Antibodies in candidate sera that bind surface proteins like SLA on porcine PBMCs can be detected with a flow-based crossmatch that mimics assays performed in human allotransplantation (4). Development of additional tools that can identify specific antigens and antibodies will be critical for success in the future. One example of such innovation may include the development of single-antigen beads that are used instead of cells to bind and screen for potentially graft-destructive antibodies in candidate sera. Nevertheless, cell-based crossmatch assays have great utility and are readily available until solid-phase assays can be developed. In effect, we know too little yet about the universe of relevant antigens and antibodies in xenotransplantation to proceed without prospective, pretransplant compatibility testing. Prospective compatibility testing is simply essential at this stage.

### Avoid overimmunosuppression.

The number of antigens that influence graft outcomes will likely be larger between species than within a species. Consequently, the optimal immunosuppression regimen for xenotransplant recipients is not known and may look quite different than successful regimens used in allotransplantation. New therapeutics may indeed be required to achieve optimal outcomes. Nevertheless, we must guard against the fallacy that greater antigen diversity will necessarily require more potent therapeutics. Even if there is a higher precursor frequency of human T and B cells specific to minor xenoantigens in the repertoire, we have incredibly powerful, established tools at our disposal to manipulate human immune responses. These include agents that deplete T and B cells (i.e., anti-thymocyte globulin and anti-CD20, respectively), block signaling through the antigen receptor (i.e., calcineurin inhibitors such as tacrolimus), and slow cell division by impairing DNA synthesis (i.e., azathioprine) or altering cell metabolism (i.e., mTOR inhibitors), to name a few. No matter how provocative the xenoantigen, our therapeutics are capable of total annihilation of recipient immune cells. Here, we must remember the two most critical lessons learned from allotransplantation: (a) our therapeutics lack tolerability and specificity, not potency, and (b) immunosuppression outside the window of tolerability for an individual patient ends in graft loss or death. In first-in-human trials, xenograft loss from rejection may be problematic as we work hard to uncover the “sweet spot” between too much and too little immunosuppression. Consequently, it is prudent to promote xenotransplantation in patient populations that have rescue modalities available should graft failure arise (i.e., dialysis), as well as to move forward with compatible xenotransplantation over incompatible transplantation to maximize safety and the likelihood of success.

### Promote success with highly selected candidates.

An overarching and urgent goal of the transplant community is to improve access to transplantation. However, xenotransplantation cannot improve transplantation access if poor outcomes ensue as a consequence of suboptimal patient selection. Well-informed, motivated research participants who are partners instead of merely participants will be key elements of successful first-in-human trials.

### Use diagnostic tests with known sensitivity and specificity.

A necessary step in the successful translation of xenotransplantation from the laboratory to the clinic will be the concurrent development of clinical-grade diagnostic tests. Although published reports herald good news and demonstrate absent transmission of porcine endogenous retrovirus (PERV) to human transplant recipients (2–4), the sensitivity and specificity of these assays is unclear. Notably, PERV tests using PCR methodology are performed routinely by veterinary laboratories, but human blood samples are not tested by veterinary laboratories. Consequently, PERV testing in all human xenotransplant recipients to date has been performed in non–Clinical Laboratory Improvement Amendments–certified (CLIA-certified) research laboratories using instrumentation, reagents, and overall workflows that may not recapitulate good clinical practice/good laboratory practice (GCP/GLP) standards in clinical laboratories. Moreover, while one study did attempt to clarify the sensitivity of the PCR-based PERV assay through serial dilution (3), this approach does not specify the limit of detection in terms of absolute molecule counts. Notably, the limit of detection in clinical diagnostic assays is always defined. This is a standard that must be achieved as clinical trials of xenotransplantation begin.

### Mind the gap.

Expertise in the management of infectious disease in immunocompromised hosts is essential for successful allotransplantation. Although much is known about the impact of specific microorganisms on pig health, this knowledge should not be considered generalizable to immunocompromised human populations. Disease caused by transmission of latent viruses in allotransplant recipients is a clear-cut example of how biology differs in immunocompetent versus immunocompromised hosts. Engagement of transplant infectious disease professionals in xenotransplantation programs conducting first-in-human trials is requisite to improve patient safety and facilitate the selection of appropriate diagnostic tests for both donors and recipients. As many transplant infectious disease specialists may lack expertise in porcine pathogens, close collaboration between infectious disease physicians, microbiologists, and veterinarians in a broad multidisciplinary team will allow the necessary exchange of information to fill the knowledge gaps.

### Ensure trial design facilitates data interpretation.

It is nearly impossible to control all the confounders in human trials that impact potential outcomes. Nevertheless, translational investigators should aim to ease interpretation of the data through trial design and/or patient selection whenever possible. While it may be easier to clinically manage a kidney xenograft recipient who is preemptively transplanted and has residual native kidney function, the transplantation in a different patient who has no residual kidney function will allow clearer interpretation of kidney xenograft function.

### Be cautious with interpretation.

Interpretation of the results of clinical trials of xenotransplantation will be challenging, given the inherent complexity of transplantation across species. Although new mechanisms of xenograft damage or destruction may indeed be discovered over the course of these trials, investigators must always consider the most likely explanation for the result. Why did the organ fail? Why did the immunosuppressed patient not do well? In transplantation, it is almost always because of infection or rejection. These diagnoses should be ruled out, not ruled in.

### Implement systems to diminish the impact of bias.

The need for an additional organ source is urgent, and with this urgency, there is pressure to provide deliverables. Although the seasoned investigators conducting trials of xenotransplantation may be immune to such pressures, reliance on trusted external consultants may help diminish the natural tendency to overinterpret experimental results, particularly in early phase I studies that involve few participants. To that end, results from trials that use blinded analysis to the extent possible will always provide the best results.

### Advocate for transparency.

The development of gene-edited pigs has been critical to the recent successful transplants in humans (2–4), and this achievement would not be possible without significant financial investment from a number of organizations. Xenotransplantation knowledge should advance in a manner that allows recoupment of investment by corporate stakeholders. Nevertheless, translational scientists must be empowered to make discoveries and identify opportunities to improve the safety and efficacy of xenotransplantation as the field matures.

### Maintain rigor.

The domestic pig has become the donor animal of choice because this species is readily available, multiplies rapidly, and has anatomy that is suitably compatible with human anatomy. Despite the advantages of using pigs as organ donors, it is not a trivial undertaking to maintain a herd free of pathogens. Pathogen-free facilities monitor for known viruses but do not address all viral pathogens. Expansion of xenotransplantation to meet the need of thousands of patients will require significant investment in such facilities, and the cost and time investment have provoked questions around the necessity of such rigor. While it may be possible to relax stringency at a point in the future when more knowledge is gained about the prevalence and impact of potential zoonotic (i.e., xenotic) pathogens, it is prudent in these early stages of xenotransplantation to maintain high expectations about the level of biosecurity at such facilities and require that xenografts for the purposes of transplantation only originate from animals maintained in such facilities.

## Conclusions

The field of transplantation is on the cusp of a breakthrough that will end the organ shortage. Such an achievement will transform not only the field of transplantation but the broader medical and scientific communities as well. This transformation will be born from science and must be kept grounded in science so that we can realize the expectations of our patients. Between our principles and our patients, we will see the way.

## Version 1 10/03/2022

Electronic publication
